# Emergency nurses´ knowledge, attitude and perceived barriers regarding pain Management in Resource-Limited Settings: cross-sectional study

**DOI:** 10.1186/s12912-019-0380-9

**Published:** 2019-11-21

**Authors:** Desale Tewelde Kahsay, Marianne Pitkäjärvi

**Affiliations:** 1 Department of Anaesthesia and Critical Care, Asmara College of Health Sciences, Mai Bela Ave, Asmara, Eritrea; 2 Metropolia University of Applied Sciences, Myllypurontie 1, PO BOX 4000, 00079 Helsinki, Metropolia Finland

**Keywords:** Pain management, Knowledge and attitude, Emergency department, Emergency nurses, Perceived barriers

## Abstract

**Background:**

Pain is a common phenomenon among emergency patients which may lead to chronic pain conditions and alteration of physiological function. However, it is widely reported that proper pain assessment and management, which is often accomplished by adequately trained nurses reduce the suffering of patients. Therefore, the aim of this study was to assess the emergency nurses´ knowledge, attitude and perceived barriers regarding pain management.

**Methods:**

A cross-sectional quantitative study design was applied to determine the nurses´ knowledge level, attitude and the perceived barriers related to pain management. Hundred twenty-six nurses from the emergency departments of seven referral hospitals of Eritrea participated in the study. Data were collected in August and September 2017. Both descriptive and inferential statistics were used to summarize and elaborate on the results.

**Result:**

In general, the knowledge level and attitude of the emergency nurses was poor. The participants’ correct mean score was 49.5%. Nurses with Bachelor’s Degree had significantly higher knowledge and attitude level compared to the nurses at the Diploma and Certificate level of professional preparation (95% CI = 7.1–16.7 and 9.4–19.1; *p* <  0.001) respectively. Similarly, nurses who had previous training regarding pain scored significantly higher knowledge level compared to those without training (95% CI =1.82–8.99; *p* = 0.003). The highest perceived barriers to adequate pain management in emergency departments were measured to be overcrowding of the emergency department (2.57 ± 1.25), lack of protocols for pain assessment (2.45 ± 1.52), nursing workload (2.44 ± 1.29) and lack of pain assessment tools (2.43 ± 1.43). There was no significant difference in perceived barriers among nurses with different demographic characteristics.

**Conclusion:**

The emergency nurses’ knowledge and attitude regarding pain management were poor. Nurses with higher educational level and nurses with previous training scored significantly higher knowledge level. This indicates the need for nursing schools and the ministry of health to work together to educate nurses to a higher level of preparation for pain assessment and management.

## Background

Pain has been the most commonly reported complaint in Emergency Departments (ED) in developed and developing countries alike [1, 2]. Researches show that once the primary causes of pain are diagnosed or identified, the main reason for which patients seek professional help is forgotten [1]. As a result, many of the patients admitted to the ED are discharged unrelieved from their pain [1, 2]. Inadequately managed pain has many consequences for the patient, family, health professionals, and society [3, 4]. Patients may have emotional reactions related to pain such as sleeplessness, anxiety and hopelessness. These reactions can be followed by unusual behaviours expressed by the patient in response to the unpleasant life experience [4]. Untreated pain has additional risks such as prolonged hospital stay, delayed recovery, and the development of chronic and persistent pain [3]. It is also known that poor analgesia leads to immobility and might also increase cardiovascular, respiratory, and gastrointestinal complications [5].

It is widely accepted that pain is a significant part of the ED nurses’ workload [6, 7]. However, literature has shown that acute pain is inadequately managed, both because of delayed administration of painkillers and under-treatment [6, 7]. In addition, it has been reported that patients in the ED might not be assessed for pain because the priority is given to the primary disease [6]. Consequently, emergency patients with low oxygen saturation and blood pressure were less likely to be considered for pain assessment [8].

The level of knowledge, along with the attitude of the ED nurses influenced the quality of care that patients receive [9]. Nurses’ foundational knowledge regarding pain has been shown to be correlated with better overall patient outcomes and satisfaction [10]**.** In contrast, deficiencies in the management of pain have been directly related to the passive participation of nurses in assessing and managing patients in pain [11]. Nurses tend to underestimate patients’ degree of suffering believing that patients self-reports about pain are exaggerated [12, 13]. In this regard, to reduce the suffering of patients, as primary caregiver, nurses must have adequate knowledge and proper attitude towards pain management [14]. Nevertheless, studies conducted in critical care, oncology, medical and postoperative care demonstrated nurses’ lack of adequate knowledge and inappropriate attitude as one of the significant barriers towards effective pain management [9, 15–17].

Although literature regarding patients´ experience of pain in ED [6.7], and their dissatisfaction with its management [18, 19] is abundant, little is known about how health professionals, particularly emergency nurses, contribute in decision making regarding pain management. As a result, evidence about emergency nurses’ knowledge and attitude with respect to pain and its management is lacking. Moreover, nurses may want to put all the necessary efforts to treat pain adequately but might be obstructed by barriers beyond their competence. In most cases, emergency nurses are dependent on orders from the physician as well as the availability of proper assessment tools and other resources to manage pain efficiently [12]. Even when some nurses have a high level of knowledge and a more appropriate attitude regarding patient pain, patients may still suffer from pain attributed to many other barriers unrelated to the scope of nursing practice.

In Eritrea, nurses have a huge responsibility in caring for emergency patients. Because physicians are not always available, nurses are the first health care professionals to face the challenge of patients pain in the ED. Therefore, in most cases, Eritrean emergency nurses initiate the management of pain even without a written order from a physician. In fact, unlike in many other countries, in most of the Eritrean hospitals, nurses who have achieved a Bachelor’s Degree and above are entitled to prescribe analgesic drugs including parenteral opioids. Despite this fact, no study has been found that has evaluated the Eritrean emergency nurses` knowledge and attitude regarding pain management. Moreover, in resource-limited settings, nurses are encountered with enormous challenges to keep their knowledge updated and only a few highly motivated and resolute individuals manage to achieve new knowledge [20]. Furthermore, Eritrean nurses’ engagement in continuous learning activities such as attending continuing education programs regarding pain assessment and management is limited due to the fact that such continuous training is seldom available.

Therefore, this study reports findings regarding the emergency nurses’ knowledge and attitude regarding pain management as well as the perceived barriers that hinder nurses from taking necessary measures to resolve patients’ suffering.

## Methods

### Research design

A non-experimental, descriptive, cross-sectional design was applied to determine the level of knowledge, attitude and the perceived barriers of nurses´ regarding pain management. A convenience sampling technique was used to recruit participants.

### Setting and sampling

After obtaining approval from the Health Research Proposal Review and an Ethical Committee of the Ministry of Health, the researchers distributed the questionnaires to the nurses working in the emergency departments of the seven national and regional hospitals of Eritrea. As these referral hospitals were serving patients referred from all health care services of the country, nurses working in these hospitals were expected to provide care for patients with intense pain. Therefore, with the limited resources and time the researchers had they target nurses working in the emergency departments of these referral hospitals in order to provide baseline evidence for further study.

The population of the current study was all 150 nurses working in the seven emergency departments of the national and regional referral hospital of Eritrea. Due to the small population size, all nurses working in the emergency departments who fulfilled the inclusion criteria were conveniently targeted to be studied. Therefore, from the 150 distributed questionnaires, hundred thirty were returned from which four were incomplete and were discarded to avoid participants with missed data. In the end, 126 (84%) questionnaires were found to be correctly filled and were entered for analysis.

### Inclusion and exclusion criteria

All nurses who were actively engaged in clinical work during the data collection period and who agreed to participate were included in the study. However, nurses who were not doing clinical work and those who were not present during the data collection period due to any reason were excluded from the study.

### Data collection

With the ethics clearance paper and data collection authorization letter from the Research and Human Resources Development, Ministry of Health, the researchers visited each hospital and explained the purpose of the study and its clinical significance to the hospital directors. After that, permission to conduct the study was obtained from each hospital director. A representative data collector from each ED was trained and had a detailed understanding of the purpose of the study. The representative data collector distributed the questionnaire to the emergency nurses who were willing to participate. Data were collected in August and September 2017. The participants returned the completed questionnaires in a sealed envelope to the representative of each hospital. Finally, the researchers collected them from each representative and opened them alone to check for completeness.

### Data collection instruments

A self-administered questionnaire consisting of three parts was used in this study. The first part asked about the demographic characteristics of the participants (seven items). The second part of the questionnaire contained thirty-five questions related to knowledge and attitude of nurses regarding pain management. The third part of the questionnaire asked the participants about the perceived barriers to pain management in EDs (twenty items).

### The nursing knowledge and attitude survey regarding pain (NKASRP) tool

NKASRP tool, developed in 1987 by Betty Ferrell and Margo McCafferty, and revised in 2014 by the same authors was used to assess emergency nurses’ knowledge and attitude regarding pain management [21]. In the current study, the NKASRP tool consisted of twenty-one true-false questions, ten multiple-choice questions and two case discussions of which each had two questions under it. NKASRP tool content validity has been established by the review of a panel of pain experts. The content of the tool is extracted from the World Health Organization, the present standard of the American Pain Society, and the National Comprehensive Cancer Network Pain Guidelines [21]. Its construct validity has been established by comparing scores of nurses at different levels of expertise [21], such as students, oncology nurses, graduate students and senior pain experts. Test-retest reliability has been set to be r > 0.80 by repeat testing, and internal consistency reliability was set at alpha r > 0.70 with items reflecting both knowledge and attitude domains. This tool has been extensively used in different languages both in developing and developed countries in other clinical contexts [16, 22–24]. Since the language of instruction in Eritrea higher institutions is English, the English version of the tool was used to collect data from the emergency nurses.

For the current study, all questions related to the management of cancer pain were removed from the questionnaire. This is because, in Eritrea, reliable oncology clinics do not exist in all the regional and national referral hospitals of the country. Subsequently, most of the drugs which are used to treat chronic cancer pain such as oral opioids do not exist in the state. Therefore, nurses are not expected to have adequate knowledge about something which does not exist and is not practised in the emergency departments. Additionally, since Vicodin (hydrocodone 5 mg + acetaminophen 300 mg) is not available in Eritrean hospitals, and there was not any other combination possible in Eritrea to replace it, one question related to Vicodin was also deleted from the questionnaire in our survey. After discussion with the pharmacovigilance unit of the ministry of health, the anticonvulsant “carbamazepine” replaced “gabapentin (Neurotin)” in one question. Additionally, modifications of some of the intravenously (IV) and intramuscularly (IM) administered opioids were required to adjust for the practical realities of Eritrean emergency departments. Therefore, from the original 40 questions, we used 35 of them.

To calculate the mean score regarding knowledge and attitude, correctly answered items were given a score of one while incorrectly responded questions were scored as zero. The total score was the sum of all correctly responded questions. In the end, to compare the result with the acceptable passing mark of 80%, the sum score for each participant was computed to 100 using SPSS version 20 as ´´sum score × 100 divided by 35″.

Even though the NKASRP tool validity and reliability has been established in previous studies [21], we also determined the validity and reliability of the modified instrument in the present study. As a result, test-retest reliability of the current study for 20 nurses in two weeks interval was 0.89 whereas the Spearman-Brown Prophecy as a measure of internal consistency was found to be 0.76 in which both were acceptable parameters [25].

When the NKASRP tool was developed in 1987, there was not a predetermined passing mark. However, in later studies, a target of 80% was set as a minimum requirement in which a score above this has been accepted as adequate knowledge and attitude regarding pain management. Therefore, referring to the recommendation of previous studies [26], nurses who scored 28 (80%) and above from the 35 knowledge and attitude questions were considered to have adequate knowledge and attitude regarding pain management.

### Tool for the perceived barriers

A standardized instrument for the collection of information regarding the perceived barriers to pain management was primarily developed in Canada and was used to collect data from the Intensive Care Unit (ICU) nurses [27]. Because the original tool was used in a different context, the researchers in the current study developed a panel of five experts (one physician. Two senior nurses from ED, and two clinical nursing instructors) to revise and establish the content validity of the modified tool. More items were added in this study to reflect specific barriers to the emergency department in an Eritrean context. The instrument was refined and re-evaluated by the five-panel of experts until a consensus was researched. Lastly, the tool was reviewed and rated by the panel of experts for content validity. In addition, the internal consistency reliability of the instrument in this study was established to be acceptable (Cronbach’s alpha = 0.81) [25].

During analysis, responses from participants were classified into 5-point scales, in which scale of 0% indicated “never,” scale of 1–25% indicated “seldom”, scale of 26–50% indicated ´´sometimes,” scale of 51–75 indicated “often,” and scale > 75% indicated “routinely.” For this study, during statistical analysis a scale of 0% was graded as 0, the scale of 1–25% as 1, the scale of 26–50% as 2, the scale of 51–75 as 3 and scale > 75% as 4. Therefore, one barrier had a possible maximum average score of four and a possible minimum score of zero while the possible total average score was expected to range from zero to 80.

### Data analysis

Each collected questionnaire was given an identification number to facilitate the capturing process of the raw data and was entered into Statistical Package for Social Sciences (SPSS) version 20 by the researchers and checked twice to assure accuracy. Data analysis was carried out using both descriptive and inferential statistics. After confirming the accuracy of the entered data, continuous variables were presented as mean, standard deviation, minimum and maximum value while nominal and categorical variables were presented as numbers and percentage.

Student T-test, analysis of variance (ANOVA), Pearson’s correlation coefficients and confidence interval were used to examine if relationship and association exist between the different demographic characteristics of the participant and their score in knowledge and perceived barriers. The significance of the difference between the two means was examined using student t-test while differences between more than two means were tested using one-way ANOVA. If statistically significant differences between groups were found when performing one-way ANOVA, Scheffee’s post hoc test was used to determine which of the groups were different from the other. Pearson’s correlation coefficient was used to determine an association between the mean score of the participants perceived barriers and their knowledge. All statistical calculations were performed using SPSS version 20 and *p*-value < 0.05 was accepted as statistically significant. To adhere to the recommendation of Ferrell and McCafferty [21], the entered data were analyzed using the percentages of total scores rather than categorising them into knowledge and attitude.

## Results

### Demographic characteristics of the participant

Demographic characteristics of the participants are shown in Table 1. The age distribution of the respondents was between 21 and 55 years with a mean age of a 28.3 ± 6.4 year. For the total 126 participants, 76 (60.3%) were males while 50 (39.7%) of them were females. Forty-nine (38.9%) of the participants were Certificate holders while 50 (39.7%) and 27 (21.4%) of the respondent were nurses with a Diploma and Bachelor’s Degree respectively. Seventy (55.6%) of the nurses had up to 48 months (4 years) of work experience while 19 (15.1%) of them had more than 96 months (8 years) of nursing work experience. The majority, 93 (73.8%) of the nurses had 36 months (3 years) and less work experience as an emergency nurse. Finally, only 40 (31.7%) of the nurses caring for emergency patients had prior training regarding pain assessment and management.

**Table 1:** Demographic characteristics of nurses participated in the study (*N* = 126)

Variable	Frequency N (%)
Age in years	
< 25	42 (33.3)
25–29	44 (34.9)
30–34	21 (16.7)
> 35	19 (15.1)
Gender	
Male	76 (60.3)
Female	50 (39.7)
Educational level	
Certificate	49 (38.9)
Diploma nurses	50 (39.7)
Bachelor of Science in Nursing	27 (21.4)
Work Experience as a nurse (months)	
≤ 24	39 (31.0)
25–48	31 (24.6)
49–72	21 (16.7)
73–96	16 (12.7)
≥ 97	19 (15.1)
Work experience as an Emergency Nurse (months)	
≤ 12	53 (42.1)
13–36	40 (31.7)
37–60	22 (17.5)
≥ 61	11 (8.7)
Previous training regarding pain management	
Yes	40 (31.7)
No	86 (68.3)

### Emergency nurses’ knowledge and attitude regarding pain management

To calculate the mean score, correctly answered items were given a score of one while incorrectly responded questions were scored as zero. The total score was the sum of all correctly responded questions. In the end, to compare the result with the acceptable passing mark of 80%, the sum score for each participant was computed to 100 using SPSS version 20 as ´´sum score × 100 divided by 35″. The mean scores and standard deviations for the total scores and percentage with the minimum and maximum score are displayed in Table 2.

**Table 2:** Means and standard deviation of the computed variable

	N	mean	SD	Minimum	Maximum
Score from 35	126	17.33	3.42	10	27
Score from 100	126	49.52	9.76	28.57	77.14

In this research, the mean total score for the knowledge-attitude survey was 49.5% in which the maximum and the minimum scores ranged from 28.6 to 77.1%, with a standard deviation of 9.76. A mean score of 80% or higher, was not achieved by any of the participants in which 57.9% of the nurses received a score of less than 50%.

Table 3 shows the number and percentages of participants correctly responded to each question. The correct response rates of each question ranged from 10 (7.9%) to 112 (88.9%). In total, only five questions had accurate response rates higher than 80% while 19 items had accurate response rates less than 50% of which six of them had response rates less than 30%. As it is indicated in Table 3, among the six least answered questions (< 30%), three of them were from the case discussions related to the assessment and management of pain in patients with the same age, surgery and level of pain but with a different way of communication and facial expression (Q 33B,32A and,32B). Two of the six least answered questions were related to the opioids side effect in which item ´30′ was related to physical independence while item ´31′ was about opioid-associated respiratory depression.

**Table 3:** Frequency of correctly answered questions (*N* = 126)

Question Items (Knowledge and Attitude)		N (%)
Least answered items (< 50%)		
32B	Your assessment for Andrew is made two hours after he received morphine 2 mg IV. Half hourly pain ratings following the injection ranged from 6 to 8 and he had no clinically significant respiratory depression, sedation, or other untoward side effects. Check the action you will take now.	10 (7.9)
30	Following abrupt discontinuation of opioid, physical dependence is manifested by the following:	17 (13.5)
33B	Your assessment, for Robert, is made two hours after he received morphine 2 mg IV. Half hourly pain ratings following the injection ranged from 6 to 8 and he had no clinically significant respiratory depression, sedation, or other untoward side effects. Check the action you will take now	23 (18.3)
32A	Andrew is 25 years old and this is his first day following abdominal surgery. He smiles at you and continues talking and joking with his visitor. He rates his pain as 8. Circle the number that represents your assessment of Andrew’s pain.	27 (21.4)
4	Patients may sleep despite severe pain	31 (24.6)
23	A 50-mg dose of IV pethidine is approximately equivalent to	33 (26.2)
28	How likely is it that patients who develop pain already have an alcohol and/or drug abuse problem?	40 (31.7)
16	If the source of the patient’s pain is unknown, opioids should not be used during the pain evaluation period, as this could mask the ability to diagnose the cause of pain correctly.	42 (33.3)
10	Elderly patients cannot tolerate opioids for pain relief	43 (34.1)
17	Anticonvulsant drugs such as Carbamazepine produce optimal pain relief after a single dose	44 (34.9)
15	Giving patients sterile water by injection (placebo) is a useful test to determine if the pain is real.	47 (37.3)
24	Analgesics for postoperative pain should initially be given	48 (38.1)
8	The usual duration of analgesia of 1–2 mg morphine IV is 4–5 h.	52 (41.3)
12	Children less than 11 years old cannot reliably report pain so clinicians should rely solely on the parent’s assessment of the child’s pain intensity.	52 (41.3)
9	Opioids should not be used in patients with a history of substance abuse	56 (44.4)
33A	Robert is 25 years old and this is his first day following abdominal surgery. As you enter his room, he is lying quietly in bed and grimaces as he turns in bed. He rates his pain as 8. Circle the number that represents your assessment of Robert’s pain	56 (44.4)
27	Which of the following describes the best approach for cultural considerations in caring for patients in pain?	57 (45.2)
11	Patients should be encouraged to endure as much pain as possible before using an opioid	58 (46.0)
1	Vital signs are always reliable indicators of the intensity of a patient’s pain	62 (49.2)
Items received 50 to 80% correct answers		
25	The most likely reason a patient with pain would request increased doses of pain medication is	65 (51.6)
3	Patients who can be distracted from pain usually do not have severe pain.	67 (53.2)
13	Patients’ spiritual beliefs may lead them to think pain and suffering are necessary.	68 (54.0)
7	Combining analgesics that work by different mechanisms (e.g., combining a NSAID with an opioid) may result in better pain control with fewer side effects than using a single analgesic agent.	69 (54.8)
26	The most accurate judge of the intensity of the patient’s pain is	74 (58.7)
6	Respiratory depression rarely occurs in patients who have been receiving stable doses of opioids over a period of months.	77 (61.1)
18	Benzodiazepines are not effective pain relievers and are rarely recommended as part of an analgesic regiment.	78 (61.9)
2	Because their nervous system is underdeveloped, children under two years of age have decreased pain sensitivity and limited memory of painful experiences.	83 (65.9)
5	Aspirin and other Nonsteroidal anti-inflammatory agents are Not effective analgesics for musculoskeletal pain	87 (69.0)
31	Which statement is true regarding opioid induced respiratory depression?	92 (73)
29	The time to peak effect for morphine given IV is	94 (74.6)
Most Answered Items (> 80)		
14	After an initial dose of opioid analgesic is given, subsequent doses should be adjusted in accordance with the individual patient’s response.	102 (81.0)
19	Narcotic/opioid addiction is defined as a chronic neurobiological disease, characterized by behaviours that include one or more of the following: impaired control over drug use, compulsive use, continued use despite harm, and craving.	102 (81.0)
22	The recommended route administration of opioid analgesics for patients with brief, severe pain of sudden onset such as trauma or postoperative	107 (84.9)
21	Sedation assessment is recommended during opioid pain management because excessive sedation precedes opioid-induced respiratory depression.	108 (85.7)
20	The term ‘equianalgesia’ means approximately equal analgesia and is used when referring to the doses of various analgesics that provide approximately the same amount of pain relief.	112 (88.9)

These items were adopted from Ferrell et al [21] with permission from the owners

### Level of knowledge and attitude in relation to selected demographic characteristics

The researchers conducted an independent t-test and one-way ANOVA to identify if differences exist between the mean score of the nurses with different demographic characteristics (Table 4). One-way ANOVA showed a significant difference in knowledge score among nurses with various educational levels; *p* <  0.001. Similarly, an independent t-test showed a significantly higher mean score of nurses who had previous training regarding pain management compared to those who had no previous training (95% CI = 1.82–8.99; *p* = 0.003). No significant differences in knowledge and attitude were found among the nurses for the other demographic characteristics such as sex, work experience, and age in which, in all cases, the *p*-value was greater than 0.05.

**Table 4:** Level of knowledge and attitude in relation to selected nurses’ demographic characteristics (*N* = 126)

Variables	N (%)	Mean ± SD	F or t	*P*-value
Educational Level			F = 28.37	< 0.001
^a^Certificate	49 (38.9)	45.5 ± 8.2		
^b^Diploma	50 (39.7)	47.9 ± 7.5		
^c^Bachelor	27 (21.4)	59.8 ± 9.1		
Age in Years			F = 0.340	0.797
< 25	42 (33.3)	49.4 ± 9.6		
25–29	44 (34.9)	48.5 ± 10.1		
30–34	21 (16.7)	50.9 ± 9.5		
≥ 35	19 (15.1)	50.5 ± 10.0		
Nursing work experience (Months)			*F = 0.662*	0.619
≤ 24	39 (31.0)	50.3 ± 9.6		
25–48	31 (24.6)	49.7 ± 9.6		
49–72	21 (16.7)	51.1 ± 11.5		
73–96	16 (12.7)	46.9 ± 8.0		
≥ 97	19 (15.1)	47.7 ± 10.1		
Emergency Nursing experience (Months)			F = 0.376	0.771
≤ 12	53 (42.1)	49.1 ± 9.8		
13–36	40 (31.7)	49.5 ± 9.8		
37–60	22 (17.5)	49.2 ± 10.5		
≥ 61	11 (8.7)	52.5 ± 8.6		
Previous Training			t = −2.98	0.003
^d^yes	40 (31.7)	53.2 ± 10.1		
^e^no	86 (68.3)	47.8 ± 9.1		
Gender			t = 0.322	0.572
Male	76 (60.3)	49.9 ± 9.8		
Female	50 (39.7)	48.9 ± 9.7		

^a^Nurse assistants with 1–2 years of training, ^b^Registered nurses with three years of training, ^c^Nurse practitioners with four years of training,^d^They had short term or long-term training regards pain either in a college or in a hospital, ^e^They had no training regarding pain

To determine if one, or any, of the three groups (nurses with certificate, diploma and/or bachelor degree) is significantly different from the other, a Post Hoc pair-wise test was conducted. As it is showing in Table 5, there were differences between the pairs of groups, nurses with bachelor’s degree being significantly different from nurses holding certificate and diploma (95% CI = 9.42–19.08 and 7.089–16.72; *p* < 0.001) respectively.

**Table 5:** Post hoc paired tests

Education Level	Mean Difference	95% CI	*p*-value
^b^Diploma vs ^a^Certificate	2.34	−1.71- 6.39	0.361
^c^Bachelor vs ^a^Certificate	14.25	9.42–19.08	< 0.001
^c^Bachelor vs ^b^Diploma	11.90	7.09–16.72	< 0.001

^a^Nurse assistants with 1–2 years of training, ^b^Registered nurses with three years of training

^c^Nurse practitioners with four years of training, CI = Confidence interval

Mean difference is significant at *p*-value < 0.001,

### Perceived barriers to effective pain management

The highest four reported perceived barriers were: overcrowding of the emergency departments 2.57 ± 1.25, lack of protocols/guidelines for pain assessment 2.45 ± 1.52, nursing workload 2.44 ± 1.29, and lack of pain assessment tools 2.42 ± 1.41 (Table 6).

**Table 6:** Perceived barriers to pain management in an emergency setting (*N* = 126)

Statement	Participants´ Response					
		Never 0%	Seldom < 25%	Sometimes 25–50%	Often 50–75%	Routine > 75%
	Mean ± SD	N (%)	N (%)	N (%)	N (%)	N (%)
Overcrowding of the Emergency Department	2.57 ± 1.25	8 (6.3)	20 (15.9)	28 (22.2)	32 (25.4)	38 (30.2)
^R^Lack of protocols/guidelines for pain assessment	2.45 ± 1.52	25 (19.8)	11 (8.7)	15 (11.9)	32 (25.4)	43 (34.2)
^R^Nursing Workload	2.44 ± 1.29	12 (9.5)	20 (15.9)	27 (21.4)	35 (27.8)	32 (25.4)
^R^Lack of availability of pain assessment tools	2.43 ± 1.43	18 (14.3)	18 (14.3)	22 (17.5)	28 (22.2)	40 (31.7)
Strict regulation of opioids	2.42 ± 1.41	13 (10.4)	26 (20.6)	26 (20.6)	17 (13.5)	44 (34.9)
^R^Lack / insufficient analgesic availability	2.34 ± 1.36	16 (12.7)	21 (16.7)	26 (20.6)	30 (23.8)	33 (26.2)
^R^Lack of protocol/ guidelines for pain management	2.25 ± 1.39	19 (15.1)	20 (15.9)	28 (22.2)	28 (22.2)	31 (24.6)
Fear of addiction towards opioids	2.17 ± 1.42	18 (14.3)	31 (24.6)	20 (15.9)	25 (19.8)	32 (25.4)
^R^Poor documentation of pain assessment and management	2.13 ± 1.37	22 (17.5)	24 (19.0)	17 (13.5)	17 (13.5)	42 (33.3)
^R^Lack of designated area for documentation	2.02 ± 1.45	28 (22.2)	21 (16.7)	23 (18.3)	29 (23.0)	25 (19.8)
Patient inability to communicate (e.g. unconscious patient)	1.98 ± 1.14	12 (9.5)	31 (24.6)	45 (35.7)	23 (18.3)	15 (11.9)
^R^Poor communication of pain and its management	1.89 ± 1.25	24 (19.0)	22 (17.5)	36 (28.6)	32 (25.4)	12 (9.5)
Insufficient analgesia dosage prescribed	1.86 ± 1.33	24 (19.0)	29 (23.0)	34 (27.0)	19 (15.1)	20 (15.9)
^R^Lack of education/ familiarity with assessment tools	1.84 ± 1.41	30 (23.8)	25 (19.9)	26 (20.6)	25 (19.8)	20 (15.9)
^R^Patient instability, e.g. unstable hemodynamic	1.79 ± 0.99	12 (9.5)	36 (28.6)	50 (39.7)	22 (17.4)	6 (4.8)
Language barriers	1.59 ± 1.24	31 (24.6)	28 (22.2)	41 (32.5)	14 (11.1)	12 (9.5)
Inadequate knowledge regarding pain management	1.58 ± 1.33	33 (26.2)	35 (27.8)	24 (19.0)	20 (15.9)	14 (11.2)
^R^Sedation interfering with pain management	1.56 ± 1.14	26 (20.6)	33 (26.2)	43 (34.2)	16 (12.7)	8 (6.3)
^R^Low priority of pain management by emergency team	1.48 ± 1.23	35 (27.8)	31 (24.6)	34 (27.0)	17 (13.5)	9 (7.1)
Patient/family requests not to give pain medications	1.21 ± 1.11	42 (33.2)	35 (27.8)	36 (28.6)	7 (5.6)	6 (4.8)

^R^Items were adopted from Rose et al [27] with permission from the corresponding author

To explore the relationship between the nurses’ perceived barriers and their demographic characteristics, independent t-test (for two means) and one-way ANOVA (for greater than two means) were used. The comparable analysis revealed that emergency nurses’ average score of the perceived barriers to pain management not to be significantly associated with their age, gender, work experience, educational level, and previous training regarding pain management (Table 7). However, Pearson’s correlation analysis revealed that emergency nurses’ perceived barriers are significantly and positively correlated with their knowledge level (*r* = 0.257, *p* = 0.004).

**Table 7:** Perceived Barriers to Pain Management in relation to Selected Nurses’ Demographic Characteristics (*n* = 126)

Variables	N (%)	Mean ± SD	F or t	*p*-value
Previous training			t = 0.521	0.604
^d^Yes	40 (31.7)	41.0 ± 12.6		
^e^No	86 (68.3)	39.8 ± 11.6		
Educational level			F = 0.996	0.372
^a^Certificate	49 (38.9)	39.6 ± 12.2		
^b^Diploma	50 (39.7)	39.3 ± 12.0		
^c^Bachelor	27 (21.4)	43.1 ± 10.5		
Emergency work experience			F = 0.065	0.978
≤ 12	53 (42.1)	40.1 ± 12.2		
13–36	40 (31.7)	39.7 ± 12.9		
37–60	22 (17.5)	40.8 ± 9.7		
≥ 61	11 (8.7)	41.2 ± 11.6		
Nursing work experience			F = 1.738	0.146
≤ 24	39 (31.0)	38.4 ± 13.98		
25–48	31 (24.6)	42.8 ± 10.7		
49–72	21 (16.7)	43.3 ± 8.3		
73–96	16 (12.7)	35.1 ± 11.8		
≥ 97	19 (15.1)	40.4 ± 11.4		
Age in months			F = 0.712	0.547
< 25	42 (33.3)	38.0 ± 13.6		
25–29	44 (34.9)	41.4 ± 11.2		
30–34	21 (16.7)	38.8 ± 10.4		
≥ 35	19 (15.1)	42.5 ± 11.0		
Gender			t = 0.013	0.909
Male	76 (60.3)	40.1 ± 12.0		
Female	50 (39.7)	40.3 ± 11.8		

^a^Nurse assistants with 1–2 years of training, ^b^Registered nurses with three years of training, ^c^Nurse practitioners with four years of training, ^d^They had short term or long-term training regards pain either in a college or in a hospital, ^e^They had no training regarding pain

## Discussion

In general, the performance of the participants on the selected aspect of knowledge and attitude was poor. Mean score of 80% or higher, which has been accepted as adequate knowledge and attitude regarding pain management [26], was not achieved by any of the participants. Nurses with a higher level of education (bachelor’s degree) scored significantly higher knowledge score than the those at the diploma and certificate level. Similarly, nurses who reportedly had previous training regarding pain scored significantly higher knowledge level than those without previous training. Findings from this study also revealed that the most commonly perceived barriers for adequate pain management in emergency departments were system related.

### Emergency Nurses’ knowledge regarding pain management

Similar to the other studies [16, 17, 23], findings from the current study revealed a severe deficit in knowledge and attitude regarding pain management.

This severe deficit in knowledge and attitude of Eritrean nurses might have arisen from the lack of attention given to pain assessment and management courses in the nursing schools. This is noticeable from the lack of sessions dedicated to pain assessment and management in most nursing school curriculums as well as the insufficient and disintegrated pain topics listed in different nursing courses. Previous studies that assessed the knowledge and attitude of the undergraduate nursing student on pain management reported a very low level of knowledge and attitude in all aspects of pain management [28–30]. The findings suggested that pain-related content of the curricula had not been enough to prepare these undergraduate nursing students to practice efficiently. Nurses who had frequent contact with patients had a higher level of knowledge and attitude regarding pain management [28]. Similarly, Aagaard et al. suggested that a curriculum with specific pain component that includes a particular model of clinical reasoning might have an influence on the development of positive attitude and belief of health care professionals towards pain [31]. Despite its worldwide high prevalence and its burden to the public, pain education has been given less priority including in medical schools. For example, a study conducted in Europe reported that only 30% of the medical schools from the representative countries had dedicated pain model in their curricula and it was compulsory only in 18% of them [32].

Another possible reason for the low scores is that pain management issues may not be a priority of policymakers in the Eritrean ministry of health. Previous researchers showed a lack of multimodal approach to pain management [33, 34], poor pain documentation, unavailability of the essential analgesic drugs, and lack of continuous medical education after graduation as the main reasons of inadequate main management after surgery [34]. In fact, in all the emergency departments, written protocols for pain assessment and management were not available during the study period; that can undeniably adversely affect pain management in the hospitals. Even though it seems to be overlooked in Eritrean hospitals, experimental studies reported that uninterrupted pain education is one of the most efficient ways to increase the knowledge, attitude and practice of nurses in assessing and managing pain [35, 36].

Therefore, revision of the school of nursing curriculums by given an emphasis on pain education, introducing continuous pain management programs [35, 36], and implementing evidence-based protocols and guidelines are suggested as a means of improving nurses knowledge and attitude and apparently improve pain management practice [37].

Similar to the finding by Bernardi et al. and Ya va et al. [17, 22], the least answered items in our study were questions related to the two case discussions. The two cases had the same age, surgery and pain intensity, but one was smiling while the other was grimacing. These issues are practical and evaluate nurses ability to proper pain assessment and management. Although 58.7% of the nurses believed that the best judge in evaluating pain should be the patient, their attitude clashed in their responses to the two cases. Similar to the previous studies, nurses’ response was influenced more by the patients’ behaviour rather than the level of pain scored. This is true because 44.4% of the nurses agreed to the level of pain the grimacing patient had while only around half of them (21.4%) agree with the level of pain the smiling patient had. Moreover, the case discussion question (32B) that asked the amount of morphine that should be administered to a smiling patient was the item that received the lowest correct responses (7.9%) while the question for a grimacing patient received 18.3% right answers. Studies conducted by Bernardi et al and Yava et al. revealed similar results in which the correct response rates for the administration of morphine for the smiling patients were 10.6 and 9.8% while for the grimacing patient were 19.1% and 23. 7% respectively.

From these case discussions we can conclude that, even though patients were identified to have severe pain, they did not receive the required amount of analgesics. In the case of the grimacing patient, 44.4% of the participants agreed that the patient was suffering from severe pain (8/10). However, despite their belief, only 18.3% of the nurses increased the dose of morphine to 3 mg which is higher than the amount of morphine administered previously. This shows that nurses had a severe knowledge deficit regarding the pharmacology of opioid analgesics. As was reported by previous studies, some of the possible reasons why nurses are hesitant to deliver a higher dose of opioids are an exaggerated fear of causing respiratory depression [26], unrealistic fear of addiction and lack of knowledge in distinguishing between dependence and tolerance [26]. One reason for the lack of knowledge about analgesic pharmacology could be the incorrect belief that analgesic drugs are in the domains of physicians’ practice and nurses have no professional duty and rights to influence physicians’ prescription [26]. But, the fact is even though the narcotic prescription is not the scope of nursing practice, nurses are responsible for determining the intervals and the amount of the opioid dose to be administered depending on the patient’s response. Therefore, without a nurses’ sound knowledge of the pharmacodynamics and pharmacokinetics of analgesics, optimal pain control seems to be impractical.

A question regarding ´´ manifestations of physical dependence following abrupt opioid discontinuation ´´ was one of the least answered (13.5%) items in this study. In Eritrea, most of the postoperative patients with pain, receive non-steroidal anti-inflammatory drugs in which the routine drug is diclofenac 75 mg intramuscular (IM) pro re nata (PRN) [34, 35]. Unarguably, this is also common practice in emergency departments. During the study period, opioid analgesics especially morphine IV/IM was often reserved for only some cases such as patients with pain secondary to myocardial infarction. Also, except for IV and IM opioids, aiming for short-term pain control, other alternatives such as the slow release opioids were unavailable. Additionally, it was reported that there were heavy regulation and strict control of opioids in all emergency departments, which was witnessed as one of the commonly occurring barriers to pain management in this study. Because of the above-detailed reasons, a manifestation of physical dependence following opioid discontinuation is not likely to be seen in the emergency departments of Eritrea. As a result, it is not a surprise that nurses had an absence of knowledge about a topic that might not have existed in the practice area.

### Level of knowledge in relation to nurses´ demographic characteristics

The present study revealed that nurses who reportedly had prior training regarding pain management had significantly higher knowledge level than nurses without previous training. In line with the present study, other previously conducted descriptive studies also reported a similar finding in which nurses who participated in any pain training program had a significantly higher mean score [17, 38]. These findings have been confirmed in other experimental studies as well. Quasi-experimental studies conducted in Iranian and Jordanian postoperative nurses demonstrated that pain management training program significantly increased both the knowledge and attitude of the participated nurses regarding pain assessment and management [35, 36].

Findings from the present study revealed that the mean knowledge score of nurses with Bachelor’s Degree was significantly higher than the nurses who had Certificates and Diplomas. This information was compatible with previous related studies [16, 17] in which nurses with higher educational background had a better knowledge related to pain management. In most Eritrean hospitals nurses who have achieved a Bachelor’s Degree work as nursing practitioners and carry considerable responsibility in emergency departments, including the prescription of analgesics for patients with pain. Despite the huge gap in the nursing school’s curriculum and lack of training in the hospitals regarding pain, it is likely that they are more motivated to read books and other sources of information. This initiative shown by the nurses might have contributed to their superior scores in knowledge and attitude towards pain management in the emergency departments. Therefore, to minimize patients suffering, effort should be made to upgrade the Certificate and Diploma nurses at to the Bachelor’s level.

In the current study, there are no significant differences found between the work experience, age, gender, knowledge and attitude of nurses regarding pain management. However, conflicting results have been reported in the literature [16, 22], yet, the majority have failed to find a meaningful association.

### Perceived barriers to effective pain management

In agreement with the findings in Taiwan [24] and northern Florida [39] emergency nurses, the top perceived barrier for pain management in Eritrea emergency departments was overcrowding of the emergency departments. Overcrowding has been known to be a significant barrier by causing delays in assessment and administration of analgesics both from the time of triage and from the time of admission [40]. During overcrowding, emergency patients may be given priority depending on the pathology of the pain rather than the severity of the pain, which further delays the administration of analgesics and prolongs patients suffering [40, 41]. Hence, patients with abdominal pain were more likely to be assessed first and receive analgesics while patients with back pain had a significant delay before they receive painkillers [6, 7]. Emergency departments triage is a system of prioritisation patients with the most life-threatening situation. Therefore, although nurses understand that every patient’s top priority during the emergency visit is a pain, unfortunately, according to the triage system, the nurses´ priority is not always a pain [39, 40].

Lack of availability of pain assessment tools, lack of protocol/ guidelines for pain management and lack of protocols/guidelines for pain assessment, where the other frequently perceived barriers reported by nurses in the current study. Since pain assessment is an integral part of adequate pain management, every emergency department must have at least one of the many validated pain assessment tools. However, it is not uncommon the intensity of pain to be judged solely by the nurses´ and other health professionals´ subjective interpretation without valid pain assessment tools. In a study that assessed the perception of pain in the emergency department demonstrated that both physicians and nurses reported significantly lower pain ratings, compared to the patients´ real report and no pain assessment tools were employed while evaluating patients´ charts [42].

Additionally, evidence-based protocols and guidelines are essential for proper evaluation and management of pain. For instance, in previous studies, introducing a new pain management protocol for patients with chronic pain was immensely helpful in reducing both the number of emergency department visits and prescription of opioids [43, 44]. A similar study evaluated the effect of a protocol-based pain therapy on time to initiation of painkillers among trauma patients [45]. Finding demonstrated that implementation of the protocol resulted in a statistically significant decrease in the average time to the starting of analgesia. However, in line with our finding, lack of protocols and guidelines on pain assessment and management have been reported as common barriers to pain assessment and management in the developing world [46, 47].

Because most of the top perceived barriers are system related, the ministry of health policymakers should work with each responsible institution to develop evidence-based protocols and guidelines regarding the assessment, documentation and management of pain in emergency situations, given the emphasis on regulations and appropriate use of narcotics. Finally, efforts should be made to increase the ratio of nurses to emergency patients so that the time allotted to each patient would increase and patients suffering in emergency departments could be minimized.

### Perceived barriers in relation to selected demographic characteristics

No statistically significant difference was found between the demographic characteristics of the nurses and the perceived barriers. However, inline to the finding by Craig and in contrast to the finding by Wang the results of the current research revealed a significant positive correlation between the average score in knowledge and the perceived barriers regarding pain management [16, 48]. The result showed that nurses with higher knowledge level regarding pain management were more barriers cautious than those who scored lower knowledge level. In general, participants in this study had an incorrect perception of their knowledge level regarding pain management. Even though they scored a very low level of knowledge and attitude, only 14 and 20% of the nurses perceived lack of knowledge regarding pain management and familiarity with pain assessment tools as a routine barrier for proper pain management respectively. However, in line with their relatively higher knowledge level regarding pain management, nurses with bachelor’s degree seemed to have a better understanding of their knowledge deficit than the nurses with lower qualifications. For instance, only 18.5% of nurses with a bachelor’s degree denied that lack of knowledge was a barrier to pain management while 30 and 26.5% of the diploma and certificate holders believed that lack of knowledge has never been a barrier to pain management.

Like many other studies, this study also has limitations. Data for this research were collected from the referral and regional hospitals of the country: which are expected to have better analgesic supplies, better opportunity for learning, and more qualified nurses with specialised physicians. Therefore, this result may not represent the smaller hospitals and other health facilities of the country, in which the knowledge deficit and the perceived barriers over there might be even worse. Additionally, knowledge doesn’t always lead to proper practice, especially if the necessary resources for adequate practice are not available. However, this study focused on the knowledge and attitude of emergency nurses regarding pain management while the valuable methods of data gathering to evaluate how nurses’ practice such as observational checklist and reviewing documents were missed.

## Conclusion

The current study showed inadequate knowledge and attitude regarding pain management. Nurses with higher educational levels and nurses with previous training regarding pain management had significantly higher knowledge level scores than those without. This gives a signal for the nursing schools to review their curriculum to increase the number of contact hours regarding pain and the hospitals to introduce a continuous pain education program to maintain the already acquired knowledge and gain new evidence regarding the modern way of pain management.

The top perceived barriers were related to emergency department overcrowding, nurses´ workload, unavailability of pain assessment and management protocols, lack of guideline regarding pain management, strict regulation of opioid, and unavailability of analgesics. All listed top barriers are system-related barriers, and therefore, nurses might not have the competence to modify them. So, policymakers of the Ministry of Health should start to move towards minimising these prominent perceived barriers.

## Supplementary information

**Additional file 1.** Questionnaire.

## Data Availability

The questionnaire used in this study is available in the article Additional file 1. The other datasets generated and analysed during the current study are not publicly available because the ethical approval and consent of participants prohibit sharing of the raw data publicly. However, when deemed necessary, the corresponding author is available to discuss any issues regarding the data requests.

## Abbreviations

ANOVA: Analysis of Variance
ED: Emergency departments
ICU: Intensive care unit
IM: Intravascular
IV: intravenous
NKASRP: Nurses knowledge and attitudes survey regarding pain
PRN: Pro re nata
SPSS: Statistical package for the social sciences
