# Supplementary material for: Emergency nurses´ knowledge, attitude and perceived barriers regarding pain Management in Resource-Limited Settings: cross-sectional study
Source: BMC Nurs. 2019 Nov 21;18:56. doi: 10.1186/s12912-019-0380-9 (PMC6873521; doi:10.1186/s12912-019-0380-9)
Supplement: Supplementary file 1 — Additional file 1. Questionnaire. [file 12912_2019_380_MOESM1_ESM.docx]

ADDITIONAL FILE 1: QUESTIONNAIRE

**NURSES KNOWLEDGE/ATTITUDE REGARDING PAIN MANAGEMENT FOR AN EMERGENCY PATIENT**

| Part I: Demographic Data | | | |
| --- | --- | --- | --- |
| 1) | Educational background: | Certificate **** Diploma **** Bachelor **** Master **** | |
| 2) | Gender: | | Male **** Female **** |
| 3) | Any previous training about pain: | | Yes **** No **** |
| 4) | Age: | | ____________ |
| 5) | Years of work experience as a nurse: | | _____________ |
| 6) | Years of work experience as an emergency nurse: | | __________**____** |
| 7) | In which Hospital are you Working:  Orotta **** Halibet **** Sembel **** Mendefera **** Keren **** Barentu **** Ghindea **** | | |

**Part II**: **Knowledge and Attitudes Survey Regarding Pain**

**True/False – Circle the correct answer.**

| T | F | 1. Vital signs are always reliable indicators of the intensity of a patient’s pain |
| --- | --- | --- |
| T | F | 1. Because their nervous system is underdeveloped, children under two years of age have decreased pain sensitivity and limited memory of painful experiences. |
| T | F | 1. Patients who can be distracted from pain usually do not have severe pain. |
| T | F | 1. Patients may sleep in spite of severe pain |
| T | F | 1. Aspirin and other Nonsteroidal anti-inflammatory agents are Not effective analgesics for musculoskeletal pain |
| T | F | 1. Respiratory depression rarely occurs in patients who have been receiving stable doses of opioids over a period of months. |
| T | F | 1. Combining analgesics that work by different mechanisms (e.g., combining an NSAID with an opioid) may result in better pain control with fewer side effects than using a single analgesic agent. |
| T | F | 1. The usual duration of analgesia of 1-2 mg morphine IV is 4-5 hours. |
| T | F | 1. Opioids should not be used in patients with a history of substance abuse. |
| T | F | 1. Elderly patients cannot tolerate opioids for pain relief |
| T | F | 1. Patients should be encouraged to endure as much pain as possible before using an opioid |
| T | F | 1. Children less than 11 years old cannot reliably report pain so clinicians should rely solely on the parent’s assessment of the child’s pain intensity. |
| T | F | 1. Patients’ spiritual beliefs may lead them to think pain and suffering are necessary. |
| T | F | 1. After an initial dose of opioid analgesic is given, subsequent doses should be adjusted in accordance with the individual patient’s response. |
| T | F | 1. Giving patients sterile water by injection (placebo) is a useful test to determine if the pain is real. |
| T | F | 1. If the source of the patient’s pain is unknown, opioids should not be used during the pain evaluation period, as this could mask the ability to correctly diagnose the cause of pain. |
| T | F | 1. Anticonvulsant drugs such as Carbamazepine produce optimal pain relief after a single dose |
| T | F | 1. Benzodiazepines are not effective pain relievers and are rarely recommended as part of an analgesic regiment. |
| T | F | 1. Narcotic/opioid addiction is defined as a chronic neurobiological disease, characterized by behaviors that include one or more of the following: impaired control over drug use, compulsive use, continued use despite harm, and craving. |
| T | F | 1. The term ‘equianalgesia’ means approximately equal analgesia and is used when referring to the doses of various analgesics that provide approximately the same amount of pain relief. |
| T | F | 1. Sedation assessment is recommended during opioid pain management because excessive sedation precedes opioid-induced respiratory depression. |

**Multiple Choice – Place a check by the correct answer.**

1. The recommended route administration of opioid analgesics for patients with brief, severe pain of sudden onset such as trauma or postoperative pain is

a. intravenous

b. intramuscular

c. subcutaneous

d. oral

e. rectal

1. A 50-mg dose of IV pethidine is approximately equivalent to:

a. Morphine 5 mg IV

b. Morphine 10 mg IV

c. Morphine 30 mg IV

d. Morphine 60 mg IV

1. Analgesics for post-operative pain should initially be given

a. around the clock on a fixed schedule

b. only when the patient asks for the medication

c. only when the nurse determines that the patient has moderate or greater discomfort

1. The most likely reason a patient with pain would request increased doses of pain medication is

a. The patient is experiencing increased pain.

b. The patient is experiencing increased anxiety or depression.

c. The patient is requesting more staff attention.

d. The patient’s requests are related to addiction.

1. The most accurate judge of the intensity of the patient’s pain is

a. the treating physician

b. the patient’s primary nurse

c. the patient

d. the pharmacist

e. the patient’s spouse or family

1. Which of the following describes the best approach for cultural considerations in caring for patients in pain?

a. There are no cultural influences in Eritrea due to similarity of the population.

b. Cultural influences can be determined by an individual’s ethnicity (e.g., Tigre are tolerant, Tigrigna are expressive, etc).

c. Patients should be individually assessed to determine cultural influences.

_d. Cultural influences can be determined by an individual’s socioeconomic status (e.g., rich individuals report more pain than poor individuals).

1. How likely is it that patients who develop pain already have an alcohol and/or drug abuse problem?

______a. < 1%

______b. 5 – 15%

______c. 25 - 50%

______d. 75 - 100

1. The time to peak effect for morphine given IV is

a. 15 min.

_b. 45 min.

c. 1 hour

_d. 2 hours

1. Following abrupt discontinuation of an opioid, physical dependence is manifested by the following:

a. sweating, yawning, diarrhea and agitation with patients when the opioid is abruptly

discontinued.

b. Impaired control over drug use, compulsive use, and craving.

c. The need for higher doses to achieve the same effect.

d. a and b

1. Which statement is true regarding opioid induced respiratory depression:

a. More common several nights after surgery due to accumulation of opioid.

b. Obstructive sleep apnea is an important risk factor.

c. Occurs more frequently in those already on higher doses of opioids before surgery.

d. Can be easily assessed using intermittent pulse oximetry.

**Case Studies**

Two patient case studies are presented. For each patient, you are asked to make decisions about pain and medication.

**Directions:** Please select one answer for each question.

1. Patient A: Andrew is 25 years old and this is his first day following abdominal surgery. As you enter his room, he smiles at you and continues talking and joking with his visitor. Your assessment reveals the following information: BP = 120/80; HR = 80; R = 18; on a scale of 0 to 10 (0 = no pain/discomfort, 10 = worst pain/discomfort) he rates his pain as 8.

A. On the patient’s record, you must mark his pain on the scale below. Circle the number that represents your assessment of Andrew’s pain.

| 1 | 2 | 3 | 4 | 5 | 6 | 7 | 8 | 9 | | 10 |
| --- | --- | --- | --- | --- | --- | --- | --- | --- | --- | --- |
| …………………………………………………………………………………………… | | | | | | | | | | |
| No pain/  Discomfort | | | | | | | | | Worst pain / Discomfort | |

B. Your assessment, above, is made two hours after he received morphine 2 mg IV. Half hourly pain ratings following the injection ranged from 6 to 8 and he had no clinically significant respiratory depression, sedation, or other untoward side effects. He has identified 2/10 as an acceptable level of pain relief. His physician’s order for analgesia is “morphine IV 1-3 mg q1h PRN pain relief.” Check the action you will take at this time.

| ________ | 1.Administer no morphine at this time. |
| --- | --- |
| ________ | 2. Administer morphine 1 mg IV now. |
| ________ | 3. Administer morphine 2 mg IV now. |
| ________ | 4. Administer morphine 3 mg IV now |

1. Patient B: Robert is 25 years old and this is his first day following abdominal surgery. As you enter his room, he is lying quietly in bed and grimaces as he turns in bed. Your assessment reveals the following information: BP = 120/80; HR = 80; R = 18; on a scale of 0 to 10 (0 = no pain/discomfort, 10 = worst pain/discomfort) he rates his pain as 8.
2. On the patient’s record, you must mark his pain on the scale below. Circle the number that represents your assessment of Robert’s pain:

| 1 | 2 | 3 | 4 | 5 | 6 | 7 | 8 | 9 | | 10 |
| --- | --- | --- | --- | --- | --- | --- | --- | --- | --- | --- |
| …………………………………………………………………………………………… | | | | | | | | | | |
| No pain/  Discomfort | | | | | | | | | Worst pain / Discomfort | |

B. Your assessment, above, is made two hours after he received morphine 2 mg IV. Half hourly pain ratings following the injection ranged from 6 to 8 and he had no clinically significant respiratory depression, sedation, or other untoward side effects. He has identified 2/10 as an acceptable level of pain relief. His physician’s order for analgesia is “morphine IV 1-3 mg q1h PRN pain relief.” Check the action you will take at this time.

| ________ | 1.Administer no morphine at this time. |
| --- | --- |
| ________ | 2. Administer morphine 1 mg IV now. |
| ________ | 3. Administer morphine 2 mg IV now. |
| ________ | 4. Administer morphine 3 mg IV now |

**PART III: PERCEIVED BARRIERS TO PAIN MANAGEMENT**

| **Please score each item as to the**  **frequency that they affect your**  **ability to assess and manage pain.** | **Never**  **(0%)** | **Seldom**  **(1-25%)** | **Sometimes**  **(26-50%)** | **Often**  **(51-75%)** | | **Routinely**  **(>75%)** |
| --- | --- | --- | --- | --- | --- | --- |
| 1. Nursing Workload |  |  |  |  |  | |
| 1. Lack of availability of pain assessment tools |  |  |  |  |  | |
| 1. Lack of education/ familiarity with assessment tools |  |  |  |  |  | |
| 1. Patient instability e.g. unstable hemodynamics |  |  |  |  |  | |
| 1. Patient inability to communicate   (e.g. unconscious patient) |  |  |  |  |  | |
| 1. Lack of protocols/guidelines for pain assessment |  |  |  |  |  | |
| 1. Low priority of pain management by emergency team |  |  |  |  |  | |
| 1. Lack of designated area for documentation |  |  |  |  |  | |
| 1. Sedation interfering with pain management |  |  |  |  |  | |
| 1. Poor documentation of pain assessment and management |  |  |  |  |  | |
| 1. Poor communication of pain and analgesic management priorities with the emergency team |  |  |  |  |  | |
| 1. Lack of protocol/ guidelines for pain management |  |  |  |  |  | |
| 1. Insufficient analgesia dosage prescribed |  |  |  |  |  | |
| 1. Strict regulation of opioids |  |  |  |  |  | |
| 1. Lack / insufficient analgesic availability |  |  |  |  |  | |
| 1. Fear of addiction towards opioids |  |  |  |  |  | |
| 1. Inadequate knowledge regarding pain management |  |  |  |  |  | |
| 1. Overcrowding of the Emergency Department |  |  |  |  |  | |
| 1. Patient/family requests not to give pain medications |  |  |  |  |  | |
| 1. Language barriers |  |  |  |  |  | |
